# Crop Growth Models: Development, Applications, Recent Advances, and Future Perspectives

**DOI:** 10.3390/plants15152395

**Published:** 2026-08-05

**Authors:** Guoan Li, Ying Wang, Yin Zhao, Zhen Liu, Shaoke Li, Xi Huang

**Affiliations:** 1 Key Laboratory of Crop Water Physiology and Drought-Tolerance Germplasm Improvement, Ministry of Agriculture and Rural Affairs, College of Agronomy, Shandong Agricultural University, Tai’an 271018, China; lgalgning@163.com (G.L.); liuzhen@sdau.edu.cn (Z.L.); 2 Bincheng Yellow River Bureau, Binzhou Yellow River Bureau, Binzhou 256600, China; 3 Shandong Key Laboratory of Eco–Environmental Science for the Yellow River Delta, Shandong University of Aeronautics, Binzhou 256600, China; wangying13@stu.sdua.edu.cn (Y.W.); lishaoke@stu.sdua.edu.cn (S.L.); 4 School of Water Conservancy, Yunnan Agricultural University, Kunming 650201, China; 2025028@ynau.edu.cn

**Keywords:** crop growth models, climate change, crop production management, remote sensing, optimization, artificial intelligence

## Abstract

Global climate change has posed a serious threat to agricultural production and food security. Crop growth models, with their excellent simulation and prediction capabilities, have become one of the important tools for guiding agricultural production and ensuring food security. This study provides a review of the research progress in crop growth models. The development history of the models is summarized into four stages, including process modeling, system simulation, model application, and algorithm expansion. According to different driving factors, the crop growth models can be categorized into solar radiation-driven, soil moisture content-driven, meteorological factor-driven, and integrated factor-driven models. In terms of the countries of development, the models mainly include those from the Netherlands, the United States, Australia, and China. Regarding applications, crop growth models are primarily applicable to adaptability assessment, agricultural resource and crop cultivation management, and climate change evaluation. Since their initial development, these models have enhanced their mechanistic nature through various approaches, such as incorporating surface mulching modules, considering the response of root water uptake to soil salt stress, and preliminarily introducing physiological regulation processes. By integrating with remote sensing technology, the spatial scale of the models has been expanded from the point scale to the regional or even global scale, and the accuracy of regional-scale yield estimation has been significantly improved through assimilation with remote sensing. Meanwhile, crop growth models have also been combined with intelligent algorithms to optimize irrigation scheduling, and to perform model parameter optimization. Looking forward, potential future research directions of crop growth models include extending the soil submodule from one-dimensional to two/three-dimensional water–heat–solute transport, moving toward a more mechanistic crop growth modeling, integration with remote sensing, and incorporating artificial intelligence. This study serves as a reference for further development and application of crop growth models, and provides technical support for the development of sustainable agriculture.

## 1. Introduction

In today’s world, humanity is facing severe challenges due to multiple issues such as ecological degradation, resource scarcity, and environmental deterioration [1]. Global warming, frequent extreme weather events, and water shortages have posed serious threats to agricultural production and food security [2,3]. Crop growth and yield information serve as a crucial foundation for national agricultural management, regulation of agricultural cropping systems, and improvement in agricultural operations, directly impacting national food security [4]. Therefore, conducting accurate and quantitative monitoring of crop growth processes and scientifically forecasting crop yields is of great significance for guiding agricultural production, ensuring food security, and promoting sustainable agricultural development.

**Figure 1:**
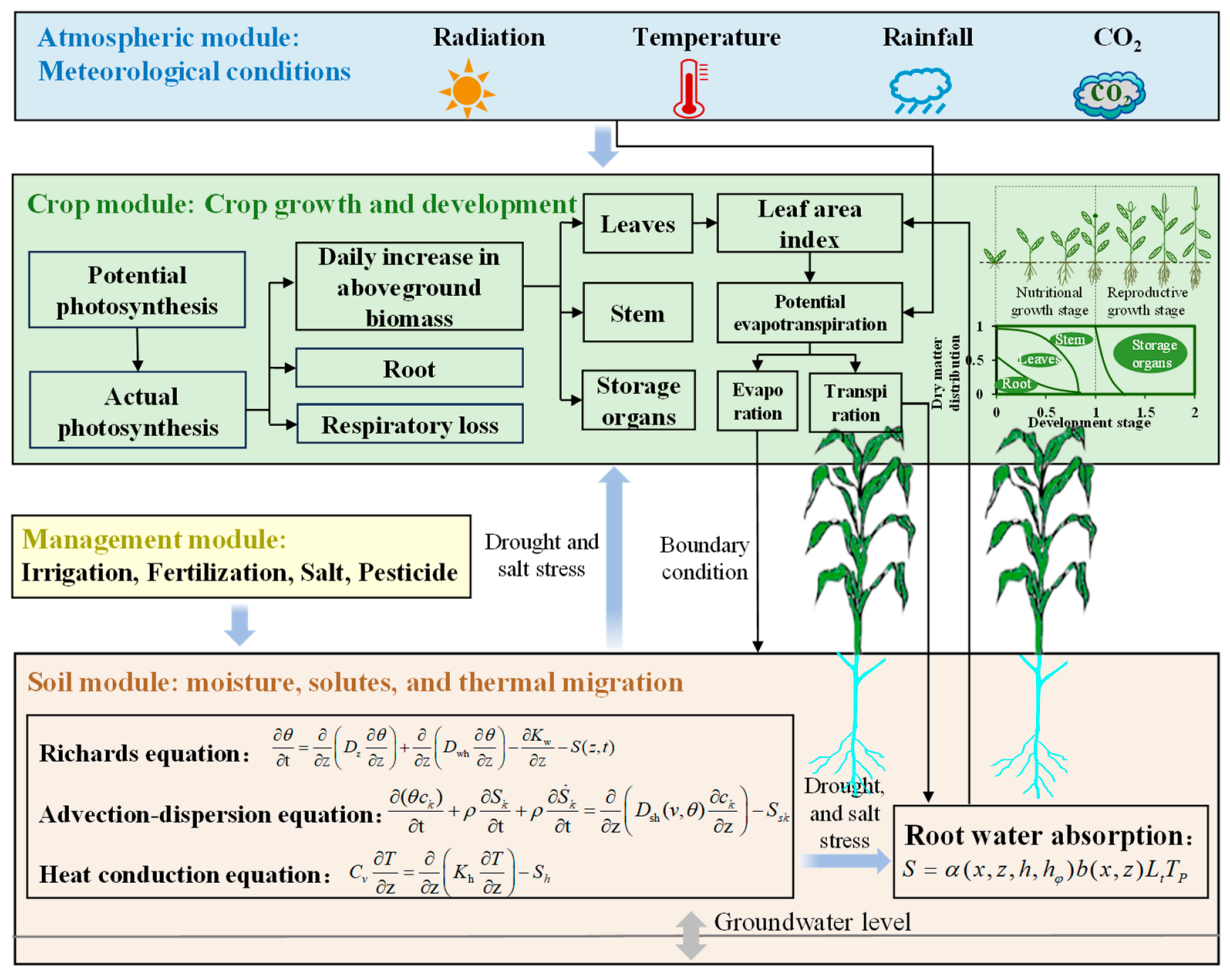
Basic framework of principles of a typical crop growth model.

Crop growth models are one of the important approaches for characterizing crop growth processes and yield formation [5]. The models use mathematical methods to describe the mechanistic processes of crop photosynthesis, respiration, transpiration, and nutrition, and they can dynamically simulate the physiological and biochemical parameters, structural parameters, and crop yield during crop growth and development at specific time steps, while also integrating various influencing factors including the soil–plant–atmosphere system [6]. The basic framework of a typical crop growth model is shown in Figure 1. Such models can quantitatively describe the effects of light, temperature, water, nutrients, field cultivation, and management practices on the balance of water, heat, salt, nitrogen, and carbon in farmland, and on crop growth, development, and yield [7]. Applying crop growth models to crop growth monitoring can assist government agencies in conducting more timely and accurate crop yield assessments and making decisions.

In recent years, crop growth models have been widely applied in agricultural science and practice, covering areas such as scientific research, agricultural resource management, policy analysis, production forecasting, climate change assessment, decision support for production management, and optimization of resource management [8]. Research on integrating these models with emerging technologies such as remote sensing, big data, and the Internet of Things has also become increasingly extensive [9]. Mainstream crop models each have their own strengths, weaknesses, and applicability scopes; therefore, it is necessary to comprehensively consider the research objectives, data availability, parameter calibration difficulty, and model suitability when conducting related simulation studies [7].

In light of this, a narrative review of the literature published from 2010 to present in the Web of Science database and China National Knowledge Infrastructure database is conducted in this study. The search employed the keyword phrases: “crop growth model”, “crop modeling”, “simulation”. The basic information, functions and applications, recent advances of crop growth models are summarized, and the current limitations and challenges are identified. The review provides scientific guidance for the further advancement of crop growth models.

## 2. Basic Information on Crop Growth Models

### 2.1. Development History

**Figure 2:**
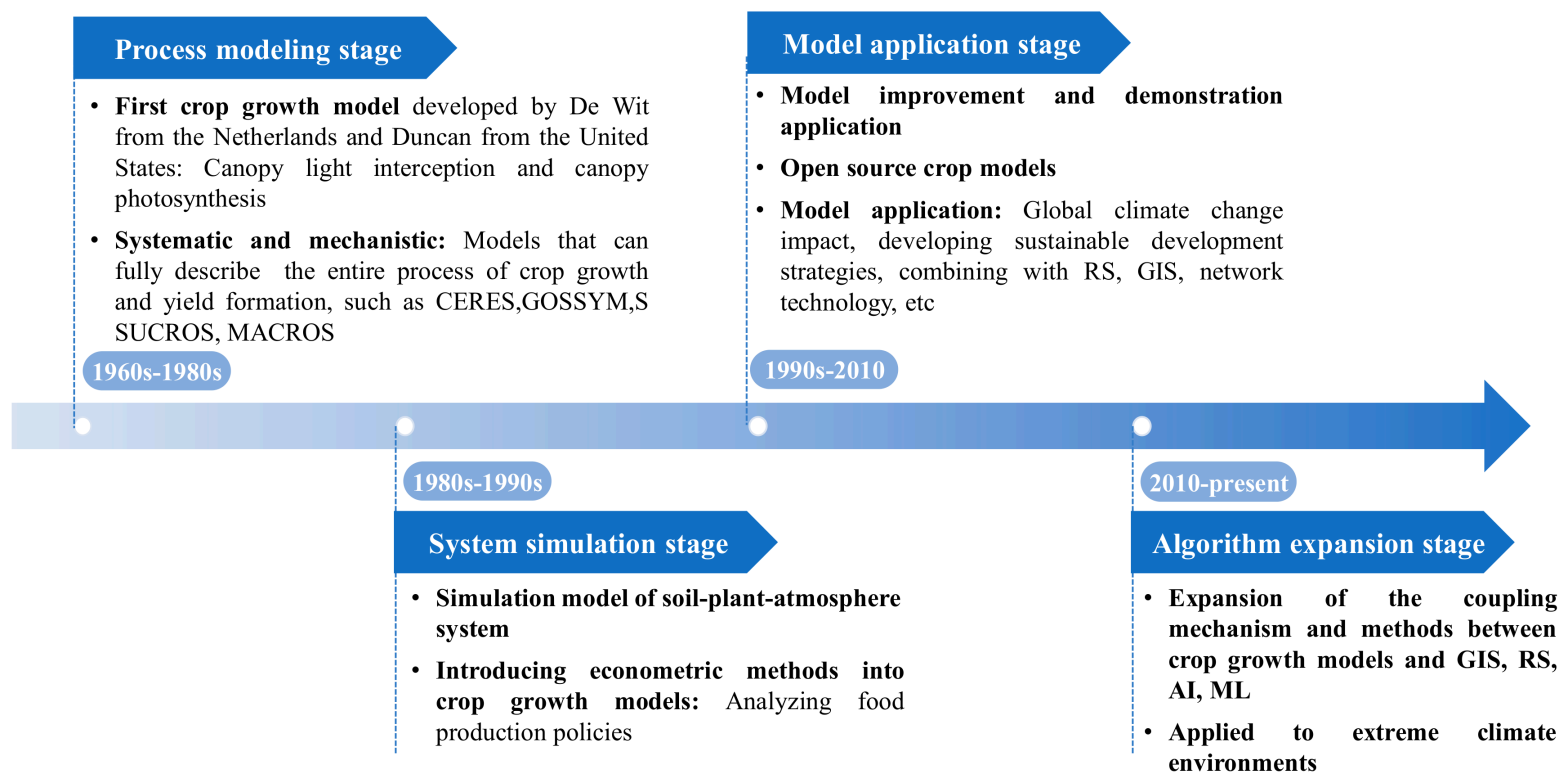
Development history of crop growth models. Note: RS, GIS, AI, ML mean remote sensing, geographic information systems, artificial intelligence, machine learning, respectively.

The development of crop growth models has evolved from conceptual models to simulation models, and from physiological and ecological processes simulation models to comprehensive cropping system simulation models. Since the pioneering work on cropping system simulation models by de Wit in the Netherlands and Duncan in the United States in the 1960s–1970s, crop simulation research has made rapid progress with the rapid advancements in systems science and computer technology, along with the continuous accumulation of knowledge in crop science, soil science, atmospheric science, and other disciplines. This progress has, in turn, made comprehensive analysis and scientific decision-making regarding cropping systems a reality. The driving forces behind the development of crop growth models primarily stem from advances in computer science and technology, the accumulation of knowledge in crop science, the quantitative requirements of management decision-making, technology transfer in agricultural extension, and the inherent uniqueness and variability of cropping systems. The international development of crop growth models can be broadly summarized into the following four main stages: process modeling, system simulation, model application, and algorithm expansion [10]. See Figure 2 for details.

### 2.2. Types of Crop Growth Models

After decades of development, scientists worldwide have developed hundreds of crop growth models, each with different theoretical foundations and characteristics. Depending on the classification method, the models can yield different categorization results. The following summarizes the classification of crop growth models based on driving factors for crop growth and development, and the countries in which they were developed.

Depending on the driving factors for crop growth and development, they can be categorized into four types: solar radiation-driven, soil moisture content-driven, meteorological factor-driven, and integrated factor-driven models [11,12]. Solar radiation-driven models primarily focus on the processes affecting dry matter accumulation resulting from photosynthesis. Commonly used solar radiation-driven crop models include DSSAT model [13], LINTUL model [14], and SAFY model [15]. The widely used soil moisture-driven models, such as AquaCrop [16] and SWAP [17], have model parameters that are heavily affected by variations in soil water content, which in turn impacts crop growth and development. Atmospheric factor-driven models exhibit pronounced effects of changes in atmospheric CO_2_, O_2_, and moisture on crop growth parameters within the models, such as SUCROS model [18] and WOFOST model [19]. Integrated factor-driven models simulate crop growth and development processes by integrating multiple branches of crop physiological simulation, allowing for unified adjustment of model input parameters and output results, such as CERES model [20] and APSIM model [21].

Based on the countries in which they were developed, crop growth models can be classified into Dutch crop models, American crop models, Australian crop models, and Chinese crop models [22]. Dutch crop models tend to be more theoretical in nature and are mostly used for validating scientific hypotheses as well as for simulating the effects of different field management practices and climate change across various regions on crop growth, development, and yield. Crop models developed in the Netherlands include SUCROS model, WOFOST model, ORYZA model, and SWAP model [23,24]. American crop models are characterized by strong practicality, operability, and specificity. They provide multiple simulation approaches to assess the impacts of environmental factors on crop growth from various perspectives, including crop physiology, soil water and nutrient dynamics, and soil organic matter. Representative models include DSSAT model, CropSyst model, and EPIC model [25,26]. Australian crop models are primarily used to promote efficient production, disaster management, and sustainable development in subtropical Australia as well as in other countries, such as APSIM model [21]. Research on crop growth models in China began in the mid-1980s. China’s first crop growth model, namely RCSODS model, was developed by Jiangsu Academy of Agricultural Sciences in 1992 [27]. In 1995, China Agricultural University proposed the WheatSM model for wheat growth and development [28]. In 2013, the National Meteorological Center of China established the CGMS-China [29]. Additionally, other crop growth models developed in China include CropGrow [30], a comprehensive crop growth model combining both mechanistic and predictive capabilities, developed by Nanjing Agricultural University, as well as the MaizeGro model [31] and the SesameGro model [32], developed by the Henan Academy of Agricultural Sciences.

## 3. Functions and Applications of Crop Growth Models

### 3.1. Adaptability Assessment

Adaptability assessment primarily involves calibrating the crop growth model using meteorological data, soil data, and physiological parameters related to crop growth processes in a specific study area to obtain the optimal variety parameters and verify the model’s simulation accuracy [33], namely model localization. Many crop growth models have been developed relatively late, and their applicability in many regions has not yet been validated; therefore, adaptability assessment is essential [8]. Adaptability assessment is the most fundamental application of crop growth models and serves as a prerequisite for their further application. In most studies, crop growth models require prior calibration and local validation before application [34]. That is, a newly developed crop growth model typically requires extensive adaptability. Even within the same study area, a large amount of measured data is needed for validation [35].

### 3.2. Resource Management and Crop Cultivation Management

Different water availability, soil fertility and crop cultivation practices can affect the soil environment, thereby influencing dynamic crop growth and ultimately impacting crop yield as well as the utilization efficiency of water and soil resources [36,37]. Therefore, it is possible to improve crop productivity and reduce negative environmental impacts by rationally allocating existing water resources and fertilizers and adjusting crop cropping systems. However, due to the randomness of water and fertilizer inputs and the diversity of crop types, planting methods, and cropping strategies, it is difficult even for professionals to determine the optimal resource input levels and crop cultivation measures [8]. As an intelligent diagnostic tool that dynamically predicts the interrelationships among crop growth and development, soil, variety, weather, and management practices, crop growth models can provide farmers with decision support for agricultural resource and crop cultivation management.

**Figure 3:**
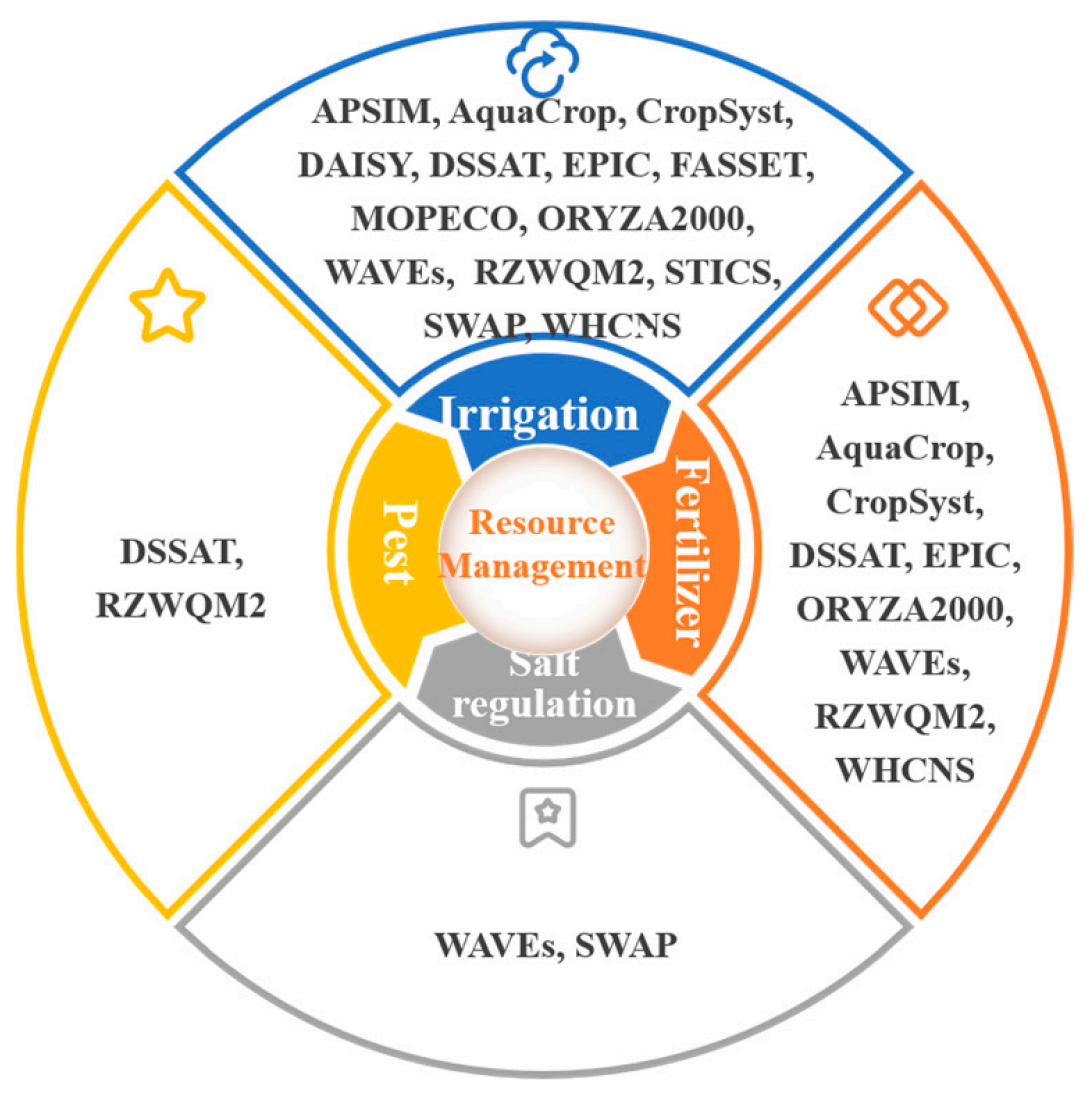
Applicability of crop growth models in agricultural resource management.

Currently, crop growth models are primarily used for water and fertilizer management, water–salt regulation, pest and disease warning, adjustment of planting time and density, and optimization of harvest time, thereby providing optimal irrigation, fertilization, salt control, pesticide application measures, and planting strategies for various specific cropping environments [38,39,40]. The process for resource and crop cultivation management is as follows: based on the adaptability assessment of the crop growth model, model parameters in the management module, such as irrigation amount, fertilization amount, soil salt content, irrigation water salinity, crop sowing time, planting density, and harvest time, are adjusted. The model is then run to evaluate crop yield and water resource use efficiency under different conditions, thereby supporting decision-making regarding agricultural resources and crop cultivation, and determining optimal resource input levels and planting measures. Nearly all crop growth models are capable of evaluating planting strategies, since planting time, plant density, and harvest time are essential input parameters in the models. Most models also include a resource management module, enabling the optimization of agricultural resource management. However, due to differences in model focus, their applicability for resource management varies. For example, AquaCrop model is primarily suitable for irrigation optimization management [41]. SWAP model focuses on irrigation and salt control management, but it is not suitable for fertilization management [42]. The DSSAT model emphasizes management of fertilization, irrigation, and pesticides [43]. The WAVES model concentrates on irrigation, salt regulation, and fertilization management [39]. Applicability of crop growth models in agricultural resource management is shown in Figure 3.

### 3.3. Climate Change Assessment

Global climate change can affect crop growth and development processes, exerting a significant impact on global agriculture [44]. Climate change assessment is the process of evaluating crop growth status in advance under future climate change conditions, such as rising temperatures and increased rainfall, so that people can take early defensive measures to cope with the adverse impacts of future climate change on agriculture [8]. Crop growth models are effective tools for predicting crop production under future climate change conditions [34].

Similarly to resource management and crop cultivation management, one application of crop growth models in climate change assessment involves adjusting meteorological parameters and running the models based on adaptability assessment to predict crop responses to various future climate conditions. Future climate change may increase crop yields [45] or decrease them [46]. This inconsistency in prediction results is primarily related to uncertainties in climate change scenarios, differences in crop types, diversity of crop varieties, and spatial variability in soil environmental conditions [47]. Since meteorological parameters are the most fundamental driving data for crop growth models, virtually all models can simulate crop responses under climate change conditions such as AquaCrop [48], APSIM [49], DNDC [50], and DSSAT [51] models.

Another application of using crop growth models for climate change assessment is the development of adaptation strategies, that is, reducing or adapting to the adverse impacts of future climate change on agriculture by adjusting planting strategies and resource utilization levels. The main measures for coping with future climate change include adjusting sowing dates [52], maintaining a certain level of initial soil moisture content [39,53], and modifying irrigation and fertilization practices [54]. Using crop models to guide adaptation measures requires that the crop models include the corresponding management modules.

## 4. Recent Advances of Crop Growth Models

### 4.1. Mechanistic Developments

Surface mulching techniques, such as plastic film mulching, straw mulching, and gravel mulching, are widely used in arid and semi-arid regions because they improve soil environment and promote crop growth [34,55]. However, when crop growth models were initially developed, surface mulching was rarely considered, and only the AquaCrop model includes simple considerations of mulching material and mulching ratio [19], which limits the application of crop growth models in farmland with surface mulching. Surface mulching modules have been added to some models such as AquaCrop, SWAP, CropSPAC, and WHCNS, accounting for the effects of surface mulching on intercepting rainfall from entering the soil, suppressing soil evaporation, temperature compensation due to plastic mulching, thermal conductivity of the film, transmission and reflection changes in shortwave and longwave radiation caused by mulching, energy emission from the film itself, and the impact of degradation rates of biodegradable plastic film on farmland water budget [37,56,57,58]. It has been proven that crop growth models that consider surface mulching factors have improved accuracy in simulating the crop growth of mulched farmland.

Some crop models, such as APSIM [21], do not inherently include a soil salinity module, which limits their application in salt-affected areas or regions where brackish/saline water is used for irrigation. To enable these models to successfully simulate crop growth under salt stress conditions, the response of root water uptake to soil salinity stress is incorporated into crop growth model. Specifically, this is achieved by establishing relationships between parameters such as the crop lower limit for water uptake, the root elongation coefficient, the root water uptake coefficient, and the electrical conductivity of the saturated soil extract (ECe) or soil chloride ion (Cl^−1^) concentration [59,60,61]. These improvements enhance the ability of the APSIM model to simulate crop biomass and yield with greater accuracy under saline conditions.

Photosynthesis is a key physiological process affecting plant growth and development, and it forms the foundation of crop yield formation [62,63]. However, traditional crop models (e.g., SWAP, APSIM, WAVEs) often neglect the physiological regulation processes of crops and pay little attention to the effects of photosynthetic physiological mechanisms on crop yield, which can reduce the accuracy of model simulations of crop growth processes. To address this limitation, researchers have integrated photosynthesis models with field-scale crop models across different scales. By evaluating the impacts of various agricultural management practices and environmental factors on crop yield from a physiologically driven perspective, crop models become more mechanistic, significantly improving the simulation accuracy of crop biomass and yield [64]. For example, Wu et al. [65] attempted to embed a daily canopy photosynthesis model (DCaPST) into the APSIM model to develop a cross-scale model, which enhanced the ability of APSIM to simulate physiology, growth and yield of sorghum and wheat under different irrigation conditions.

### 4.2. Model Scale Improvement Through Assimilation with Remote Sensing

Crop growth models can simulate daily or even second-by-second soil water, salt, heat, nitrogen, carbon, and crop growth status, meaning that crop models have achieved temporal continuity from the time of their development [8]. In terms of spatial scale, except for the DayCent model, which is suitable for assessing crop productivity from regional to global scales, most crop models are applicable to point-scale simulations [8]. This introduces errors and uncertainties in crop growth simulation and yields prediction from regional to global scales, as the spatial heterogeneity of near-surface environmental conditions leads to biases in the spatial distribution of meteorological, soil, and crop parameters within the crop growth models [66]. Therefore, further improvement in the spatial scale of crop models is needed.

Remote sensing inversion technology is the process of using data such as reflectance and backscattering coefficient obtained from satellite sensors to invert the biophysical or environmental parameters of crops through physical or empirical models [67]. Extensive research has been conducted on inversion methods for key parameters such as leaf area index and soil moisture [68,69,70]. Although remote sensing inversion methods can obtain large-scale surface information, they are difficult to decipher the underlying mechanisms of crop growth [71]. The spatial advantages of remote sensing make it possible for crop growth models to achieve spatiotemporal continuity, which requires the combination of crop growth models and remote sensing technology to achieve complementary advantages between remote sensing and crop growth models. In recent years, data assimilation techniques have been used to combine remote sensing inversion observation data with crop growth models. Specifically, by continuously updating model states and parameters, data assimilation techniques effectively correct model simulation biases, reduce accumulated errors, and thereby mitigate parameter uncertainty and spatial heterogeneity [67,72]. This improves the accuracy of regional crop growth and yield simulations, providing technical support for achieving precision management in large-scale agriculture [73].

**Figure 4:**
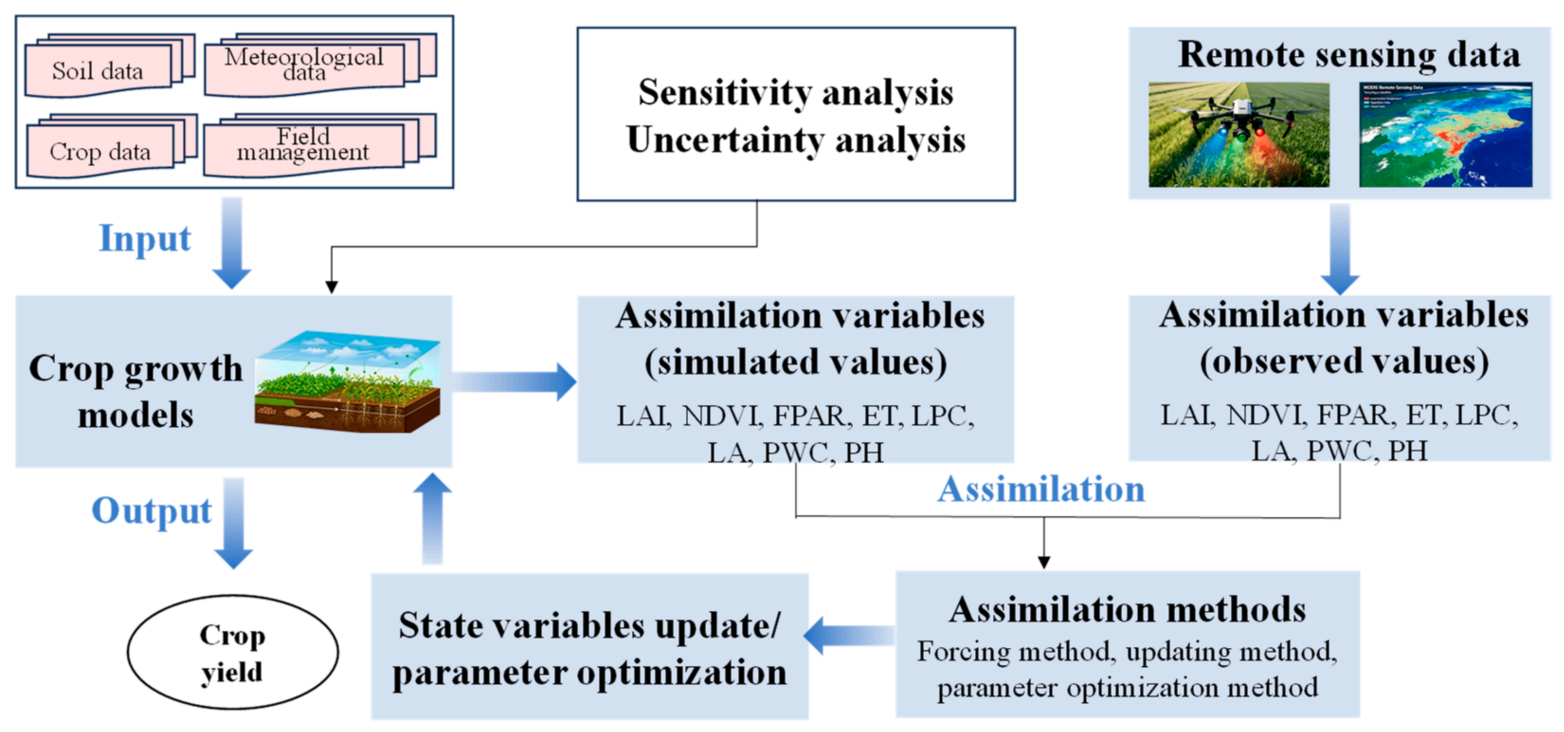
Schematic diagram of assimilation system between remote sensing and crop growth model. Note: LAI, NDVI, FPAR, ET, LPC, LA, PWC, and PH mean leaf area index, normalized difference vegetation index, fraction of photosynthetically active radiation, evapotranspiration, leaf pigment content, leaf angle, plant water content, and plant height.

Coupling crop growth models with remote sensing data requires the selection of appropriate assimilation variables, assimilation methods, crop growth models, and remote sensing data (Figure 4). Assimilation variables are data shared by both crop growth models and remote sensing retrievals, serving as the bridge between the two. The main assimilation variables include leaf area index (LAI), normalized difference vegetation index (NDVI), fraction of photosynthetically active radiation (FPAR), evapotranspiration (ET), leaf pigment content (LPC), leaf angle (LA), plant water content (PWC), and plant height (PH) [8,74]. Assimilation methods integrate assimilation variables from both sources to obtain more accurate and comprehensive information. The primary data assimilation methods include forcing, updating, and parameter optimization. The forcing method directly replaces the initial state variables of the crop growth model with remote sensing observations. The updating method continuously corrects the state variables simulated by the model using remote sensing observations [68]. The parameter optimization method adjusts the internal parameters of the crop growth model to minimize the discrepancies between the assimilated variables [67]. Their respective characteristics are presented in Table 1. Regarding the selection of crop growth models, due to its fully open-source code and relatively simplified soil module, the WOFOST model is the most widely used crop growth model [75], followed by the DSSAT model [76]. Other crop growth models that have been combined with remote sensing data include AquaCrop [77] and SWAP [74]. For remote sensing data, depending on the study area and objectives, small-scale assimilation more frequently uses UAV remote sensing data, Sentinel, Landsat series, and HJ-1A/B data, while large-scale assimilation predominantly uses MODIS remote sensing data [8]. The observation operator serves as the core bridge connecting crop models with remote sensing observations. It maps model state variables to the observation space, thereby resolving the inconsistency in physical meaning between simulation variables and remote sensing observation signals. Depending on the application scenario, observation operators can take forms of varying complexity: (1) direct comparison operators, which assume that model outputs and remotely sensed retrieval products can be directly compared [75]; (2) physical model operators, such as the PROSAIL radiative transfer model, which directly convert canopy parameters into spectral reflectance and can avoid cumulative errors inherent in the retrieval process [78]; and (3) statistical or machine learning operators, which refer to empirical relationships established between vegetation indices and model states [79].

Integrating remote sensing data into crop models through data assimilation techniques has proven to be an effective approach for improving the accuracy of regional crop yield estimation [80,81]. Table 2 presents the configurations and validation results of crop model assimilation systems in some representative studies. Differences among studies in terms of assimilated variables, assimilation methods, crop models, and remote sensing data selections lead to considerable fluctuations in the efficiency and accuracy of yield estimation, with the root causes primarily lying in the uncertainties introduced at each of the aforementioned stages. Specifically, regarding assimilated variables, various remote sensing products, such as LAI, normalized NDVI, ET, PH, and soil moisture, exhibit notable differences in spatial coverage, temporal resolution, sensitivity, saturation characteristics, and retrieval errors. For instance, NDVI is prone to saturation under high biomass or high vegetation cover conditions, making it insensitive to changes in canopy structure during the mid-to-late growth stages of crops, thereby introducing estimation biases [82]. LAI and soil moisture are simultaneously influenced by multiple interacting factors, including soil, leaf, and canopy properties, which increase the difficulty of their accurate quantification [83]. At the level of assimilation methods, different assimilation algorithms inherently possess distinct mathematical mechanisms and underlying assumptions. For example, parameter optimization methods entail substantial computational costs and are prone to becoming trapped in local optima, while filtering methods suffer from filter divergence and perform poorly when handling strongly nonlinear relationships, directly leading to discrepancies in yield estimation accuracy [84]. Uncertainties at the crop model level mainly manifest as model parameter settings, model structure, and spatiotemporal heterogeneity arising from regional cropping patterns, field management practices, climatic backgrounds, and soil physical properties [84]. Meanwhile, the spatiotemporal resolution of remote sensing data, instrumental errors of satellite sensors, and representativeness errors introduced by retrieval models can all potentially affect the final yield estimation results [85]. Uncertainties inherent in the observation operator itself, including parameterization errors and scale mismatches, can also significantly compromise assimilation performance [86]. In addition, implementation details such as regional-scale grid partitioning and assimilation time windows are also considered important factors affecting the accuracy of yield estimation [87].

Researchers have employed various strategies to reduce uncertainties, including improving assimilation algorithms, hybrid assimilation approaches, multi-source remote sensing fusion, multi-model coupling, and model parameter sensitivity analysis combined with parameter optimization. For example, combining four-dimensional variation (4D-VAR) with other optimization algorithms, such as the SCE-UA algorithm [92], not only improves the accuracy of crop yield estimation but also accounts for both observation and model errors simultaneously. The particle swarm optimization (PSO) algorithm has been combined with the Hellinger to reduce biased estimation [93]. Studies have demonstrated that state variables derived from multi-source remote sensing can be assimilated into crop models, which reduces input data uncertainties and enhances the accuracy of crop yield estimation [94,95]. Coupling multi-source remote sensing observations offered advantages such as large-area monitoring capability, short revisit intervals, and high data resolution, providing richer spatiotemporal datasets [96], thereby overcoming the issues of long revisit cycles and susceptibility to adverse atmospheric conditions (e.g., cloud cover) associated with single-source remote sensing observations [97]. Multi-model coupling improves simulation accuracy and system stability by integrating the strengths of individual models while also reducing model operational costs. For example, after coupling CASA and WOFOST, the resulting CASA-WOFOST inherits the fast computational speed of the former and the process-based mechanistic nature of the latter, thereby improving both the accuracy and efficiency of crop yield estimation [98].

### 4.3. Coupling with Intelligent Algorithm

Coupling crop growth models with optimization algorithms to optimize irrigation scheduling has become an important research direction in the field of efficient utilization of agricultural water resources in recent years. Irrigation scheduling optimization refers to the process of scientifically determining irrigation timing, irrigation frequency, irrigation quota, and total irrigation quota based on crop growth and development patterns, soil water movement dynamics, and meteorological conditions under specific regional and agricultural production conditions, using mathematical models and system analysis methods to achieve multiple objectives such as water conservation, yield increase, high efficiency, and sustainability [99]. Crop growth models are effective tools for optimizing irrigation scheduling. However, when used alone, they can only answer “what if” questions, meaning that the optimal irrigation schedule is only optimal under the scenarios specified by the user, rather than being globally optimal [100]. This necessitates the integration of crop growth models with optimization algorithms to build simulation–optimization-based irrigation scheduling optimization models. Subsequently, optimal solution sets are sought based on specific objectives, and finally, decision-making methods are used to select among the optimized results.

The process of using crop model optimization algorithms for irrigation scheduling decision-making involves determining the crop growth model, optimization objectives, optimization solution methods, and decision-making methods [99]. The AquaCrop model, due to its simple structure, low parameter requirements, low computational complexity, and reasonable accuracy, has become the most widely used model in irrigation scheduling optimization [101], followed by the DSSAT model [102]. Optimization objectives typically include one or more of the following: maximizing crop yield, minimizing irrigation water amount, maximizing irrigation water productivity, maximizing water use efficiency, and maximizing profit [103]. Optimization solution methods include dynamic programming, fuzzy optimization algorithms, genetic algorithm optimization, simulated annealing, particle swarm optimization, ant colony algorithms, and fish swarm algorithms [104]. Among these, the NSGA-II algorithm is the most popular, especially in multi-objective optimization models [105]. Decision-making methods mainly include comprehensive evaluation approaches such as the analytic hierarchy process (AHP), entropy weight (EW), principal component analysis (PCA), gray relational analysis (GRA), and the technique for order preference by similarity to an ideal solution (TOPSIS) [104].

The crop model has multiple parameters and strong spatiotemporal variability, necessitating parameter calibration prior to model application. To overcome the drawbacks of traditional trial-and-error methods, which are time-consuming, labor-intensive, and heavily dependent on researcher subjectivity, crop models have been coupled with parameter optimization algorithms to improve the efficiency of parameter calibration and enhance model simulation accuracy [106]. Commonly used parameter optimization methods include parameter estimation (PEST), genetic algorithms (GAs), machine learning algorithms, universal inverse code (UCODE), and shuffle complex evolution method (SCE-UA) [106,107]. These methods have been successfully applied to optimize soil hydraulic parameters, solute transport parameters, crop model parameters, and others [108]. Among these, PEST and GA are the most widely used. PEST employs an improved nonlinear least-squares optimization algorithm, while GA is an optimization and heuristic search technique that can achieve the global optimal solution through appropriate selection of structure, parameters, and iteration numbers. For example, the PEST program was employed to calibrate soil hydraulic parameters and crop growth parameters for the H2DSWAP model, achieving rapid and efficient optimization of selected model parameters [4]. AquaCrop model parameters were calibrated using an elitist genetic algorithm and the differences between intelligent algorithms and manual trial-and-error methods were evaluated, which concluded that the genetic algorithm offered advantages in terms of global optimization speed and accuracy [109].

## 5. Limitations of Crop Growth Models and Future Research Directions

### 5.1. Extending the Soil Submodule from One-Dimensional to Two/Three-Dimensional Water–Heat–Solute Transport

Almost all of the crop growth models mentioned above in this study are one-dimensional. It can be seen that, whether in terms of model functions or development, one-dimensional coupled models of soil water, heat, salt, and nitrogen transport with crop growth are relatively mature and widely applied. However, in actual agricultural production, especially with the continuous promotion of drip irrigation technology in recent years, one-dimensional crop growth models can no longer meet the demands of real farmland conditions. Although one-dimensional models are still applied to farmlands with three-dimensional soil water, heat, salt, and nitrogen transport under drip irrigation [110], the reliability of the model simulation results requires further verification.

The HYDRUS-2D/3D model is a relatively mature two-dimensional/three-dimensional model for soil water, heat, salt, and nitrogen transport, and it lacks a dynamic crop growth module. Currently, the HYDRUS-2D/3D model has been coupled with several crop growth models, for example, HYDRUS-2D/3D with the SWAP model and EPIC model, to assess soil water and salt transport and crop growth processes under drip irrigation or mulched drip irrigation conditions [4,111,112]. However, such coupled models are still in their early stages and require further development and application.

### 5.2. Toward a More Mechanistic Crop Growth Modeling

As mentioned in Section 4.1 of this study, the APSIM crop growth model has already been coupled with the daily canopy photosynthesis model DCaPST, achieving physiological mechanistic representation of crop growth processes. Nevertheless, this integrated approach remains at an early exploratory stage, and several important knowledge gaps remain to be addressed. First, the applicability of this coupled model under other crop types, climatic conditions, and soil conditions has yet to be systematically evaluated. Second, the generalizability of the DCaPST model coupling with other crop growth models has not yet been demonstrated. Third, the DCaPST model estimates stomatal conductance using the Penman Monteith empirical formula, which limits its capacity to represent the combined effects of stresses such as drought or salt stress, and biological factors, on stomatal behavior. Finally, further development of more mechanistic physiological regulation processes is necessary.

### 5.3. Integration with Remote Sensing

Significant progress has been made both domestically and internationally in remote sensing retrieval and data assimilation technologies, while many challenges remain. In terms of remote sensing retrieval, improving accuracy under complex surface conditions, enhancing algorithmic computational efficiency, and fusing multi-source data remain research priorities. In terms of data assimilation, the performance of currently used filtering assimilation algorithms is highly dependent on the model parameters’ calibration. Although they can reduce simulation uncertainty by correcting key state variables (e.g., LAI, soil moisture), they are unable to correct biases in cultivar parameters that directly affect grain development and are independent of other states; moreover, they may even amplify errors when the model calibration exhibits substantial deviations [113]. Variational methods that adjust only model parameters focus on parameter optimization but are susceptible to model structural errors. When encountering anomalous disturbances not described by the model, the recalibrated parameters not only fail to improve prediction accuracy but also may instead lead to worse results due to abnormal observations [114].

Joint state–parameter estimation can compensate for the respective shortcomings of the two traditional approaches of “updating states only” and “adjusting parameters only” [84]. Therefore, the assimilation strategy of joint state–parameter estimation represents a promising direction, with the augmented EnKF (AEnKF) being a typical example of this approach. Hu et al. [115] compared the performance of the AEnKF and the EnKF in correcting WOFOST-SWAP model predictions as well as the influence of state vector construction based on Observing System Simulation experiments (OSS); their results indicated that the AEnKF can alleviate the inconsistency between parameters and state vectors. In particular, updating the development stage can avoid the negative effects of “phenological drift”.

However, the assimilation strategy of joint state–parameter estimation still faces significant challenges. First, the sparsity of remote sensing observations makes it difficult for many parameters, especially those that significantly affect state variables only during specific phenological phases to effectively converge to their true values [84]. Second, phenological parameters exhibit strong spatiotemporal variability owing to environmental and field management influences, and directly updating them disrupts the internal temporal consistency of the model. Countermeasures such as restarting the EnKF or refreshing phenological parameters are either computationally expensive or difficult to implement in complex models [88]. Therefore, although current joint estimation methods are beneficial, they also carry risks. In the future, more robust algorithms need to be developed, which are capable of adapting to the specific structure of crop growth models and respectively accommodating the distinct characteristics of time-varying parameters, time-invariant parameters, and phenological parameters, so as to achieve truly effective joint state–parameter estimation.

### 5.4. Incorporate Artificial Intelligence

Artificial Intelligence (AI) technologies, encompassing machine learning, deep learning, and reinforcement learning, are rapidly permeating all aspects of agricultural production. In the field of crop modeling, these technologies are playing an increasingly important role in data preprocessing, parameter calibration, parameter optimization, uncertainty analysis, result interpretation, and yield prediction [116]. For example, Yu et al. [117] employed two ensemble learning algorithms—Random Forest and Gradient Boosting Trees—to screen and optimize sensitive parameters in the growth process of dryland spring wheat based on APSIM model, which reduced the root mean square error of yield simulations by approximately 50%.

In addition to parameter optimization within models, AI technologies can also empower crop modeling by improving the quality of external observational data. Rana et al. [118] developed an automated segmentation pipeline based on multitemporal UAV aerial imagery and ortho-mosaic images, integrating YOLOv8, Grounding DINO, and the Segment Anything Model (SAM) to achieve automatic annotation and instance segmentation of individual cauliflower crop rows. From the resulting segmentation masks, the study extracted pixel counts for each crop row on different dates, subsequently calculating relative growth rates and conducting time-series analyses to quantify crop growth dynamics over time. This study provides a practical paradigm for leveraging advanced computer vision techniques to automatically extract spatially explicit crop growth indicators from repeated UAV observations; the extracted indicators can be further utilized for crop model calibration, validation, or data assimilation. In general, AI technologies can provide high-resolution, spatiotemporally continuous observational inputs to the model, while crop models are dedicated to simulating the intrinsic biological mechanisms of crop growth, with the two approaches complementing each other functionally.

Therefore, constructing a hybrid modeling framework that integrates “mechanistic models + AI + remote sensing,” using remote sensing time-series data to drive the updating of key parameters and the correction of state variables in crop growth models, merging multi-dimensional remote sensing observations such as evapotranspiration, soil moisture, and phenological information, combining deep learning algorithms to extract temporal features, and incorporating advanced data assimilation and optimization algorithms, is expected to significantly enhance the model’s adaptability and predictive accuracy for crop growth processes under extreme climate and other disaster-induced stress conditions. Currently, this direction is still in its preliminary exploratory stage, and AI technologies still face numerous challenges in terms of data security and reliability, computational resource availability, and model interpretability, which urgently require systematic investigation in future research.

## 6. Conclusions

This study reviews the research progress in crop growth models. First, development history of the models is reviewed, and they are classified based on the countries where they are developed and the driving factors for crop growth and development. Second, functions and applications of crop growth models from three aspects are elaborated: adaptability assessment, agricultural resource and crop cultivation management, and climate change assessment. Third, recent advances of the models are summarized in terms of model mechanisms, model scale improvement through assimilation with remote sensing, and coupling with intelligent algorithms. Finally, four potential future research directions of crop growth models are identified, including extending the soil submodule from one-dimensional to two/three-dimensional water–heat–solute transport, moving toward a more mechanistic crop growth modeling, integration with remote sensing, and incorporating artificial intelligence. Further improving the capabilities of these models will help to more effectively address complex issues in agricultural systems and support the sustainable development of global food production.

## Figures and Tables

**Table 1:** Classification of assimilation algorithms and their advantages and disadvantages.

Category	Advantages	Disadvantages	Examples
Forcing method	Relatively simple principle	Overly reliant on external observations; does not account for errors in the observation model itself	-
Updating method	Consider both external observations and uncertainties in model simulations	Error accumulation; simulation has abrupt change points, and carbon balance, water balance, and nutrient balance are no longer closed	Ensemble Kalman Filter (EnKF), Particle Filter (PF)
Parameter optimization method	Reduce error accumulation and propagation	High number of iterations; low computational efficiency	Four-dimensional variation (4D-VAR), particle swarm optimization (PSO), genetic algorithm (GA), simulated annealing (SA)

**Table 2:** Configuration and validation results of crop model assimilation systems from some representative studies.

Crop Models	Sensor and Platform	Assimilated Variable	Observation Operator	Assimilation Method	Spatial/Temporal Resolution	Validation Strategy	Improvement over the Non-Assimilated Baseline	Reference
WOFOST	UAV, multispectral imaging system	LAI	Direct comparison	EnKF	-/-	RMSE, NRMSE, MRE	Reduce NRMSE of yield from 12% to 4%	Fu et al. [75]
DSSAT	Satellite-based, microwave sensors	Surface soil moisture	Direct comparison	AEnKF	0.25°/1 d	RMSE, R, Eff	Reduce RMSE of soil water content from 0.040–0.053 to 0.031–0.040	Zhou et al. [76]
WOFOST	Sentinel-2	LAI	Multi-Output Gaussian process regression model	ENKF/Two-step Bayesian inference on state and parameter	-/8 d	R^2^, RMSE, MAPE	Reduce RMSE from 1395 kg/ha to 1112 kg/ha	Song et al. [88]
APSIM	ESA CCI, SMAP + HydroBlocks, SMAP-Sentinel1 (1 km, 3 km)	Soil moisture	PROSAIL model	EnKF combined with Miyoshi algorithm	0.25°/1–2 d, 30 m/1–3 d, 1 km/12 d, 3 km/12 d	RMSE, NRMSE, R^2^	Reduce RMSE of yield by 17.2%–23.0%	Marissa et al. [78]
CAMM	MODIS	LAI	Direct comparison	EnKF, 4DVar	500 m/8 d	r, bias, NRMSE	Reduce NRMSE of yield from 11.5% to 8.4%	Han et al. [89]
WOFOST	UAV	LAI	Direct comparison	EnKF	50 m/-	RMSE, MSE, NRMSE	Reduce RMSE of yield from 392 to 215 kg/ha	Guo et al. [90]
DSSAT	Handheld GreenSeeker	LAI, PNA	Direct comparison	MCMC	-/10 d	R^2^, RMSE, RE	Reduce RMSE of yield from 13.8 kg/ha to 7.3 kg/ha	Chen et al. [91]
RSCM	Handheld multispectral radiometer	LAI	Machine learning regression model	Powell algorithm	-/1 d	RMSE, MAE, NSE	Rice: Reduce RMSE of LAI from 0.70 to 0.28, improve NSE from 0.29 to 0.88 Soybean: Reduce RMSE of LAI from 1.03 to 0.72, improve NSE from 0.49 to 0.75	Ko et al. [79]

Note: WOFOST, WOrld FOod Studies; DSSAT, Decision Support System for Agrotechnology Transfer; APSIM, Agricultural Production Systems sIMulator; CAMM, Chinese Agricultural Meteorological Model; RSCM, Remote Sensing-Integrated Crop Model; UAV, unmanned aerial vehicle; ESA CCI, European Space Agency Climate Change Initiative; SMAP, Soil Moisture Active Passive; LAI, leaf area index; PNA, plant nitrogen accumulation; EnKF, Ensemble Kalman Filter; AEnKF, Augmented Ensemble Kalman Filter; MCMC, Monte Carlo Markov Chain; RMSE, root mean square error; NRMSE, normalized root mean square error; MRE, mean relative error; R, correlation coefficient; Eff, effectiveness; MAPE, mean absolute percentage error; R^2^, determination coefficient; r, Pearson correlation coefficient; MSE, mean squared error; RE, relative error; MAE, mean absolute error; NSE, Nash–Sutcliffe efficiency coefficient.

## Data Availability

No new data were created or analyzed in this study. Data sharing is not applicable to this article.
